# Combining Feature Selection Techniques and Neurofuzzy Systems for the Prediction of Total Viable Counts in Beef Fillets Using Multispectral Imaging

**DOI:** 10.3390/s23239451

**Published:** 2023-11-27

**Authors:** Abeer Alshejari, Vassilis S. Kodogiannis, Stavros Leonidis

**Affiliations:** 1 Department of Mathematical Science, Princess Nourah Bint Abdulrahman University, Riyadh 11671, Saudi Arabia; aaalshejari@pnu.edu.sa; 2 School of Computer Science & Engineering, University of Westminster, London W1W 6UW, UK; 3 Consulting & Systems Integration, Netcompany-Intrasoft, GR-57001 Thessaloniki, Greece; stavros.leonidis@netcompany.com

**Keywords:** neural networks, ensemble systems, fuzzy logic, beef, total viable counts, regression, multispectral imaging, machine learning

## Abstract

In the food industry, quality and safety issues are associated with consumers’ health condition. There is a growing interest in applying various noninvasive sensorial techniques to obtain quickly quality attributes. One of them, hyperspectral/multispectral imaging technique has been extensively used for inspection of various food products. In this paper, a stacking-based ensemble prediction system has been developed for the prediction of total viable counts of microorganisms in beef fillet samples, an essential cause to meat spoilage, utilizing multispectral imaging information. As the selection of important wavelengths from the multispectral imaging system is considered as an essential stage to the prediction scheme, a features fusion approach has been also explored, by combining wavelengths extracted from various feature selection techniques. Ensemble sub-components include two advanced clustering-based neuro-fuzzy network prediction models, one utilizing information from average reflectance values, while the other one from the standard deviation of the pixels’ intensity per wavelength. The performances of neurofuzzy models were compared against established regression algorithms such as multilayer perceptron, support vector machines and partial least squares. Obtained results confirmed the validity of the proposed hypothesis to utilize a combination of feature selection methods with neurofuzzy models in order to assess the microbiological quality of meat products.

## 1. Introduction

The food industry is constantly concerned about the quality of their products, as consumers always expect to find food products with excellent quality but at affordable prices [1]. In order to meet such consumers’ expectations, food industry requires some suitable detection methods to guarantee the quality of their products. Muscle foods, which include both meat and poultry, have been considered an integral part of the human diet for many years. Beef is one of the widely consumed commercial muscle foods throughout the world and there is an expectation that this trend will be increased based on consumers’ food preferences, due to health, nutrition and diet factors [2]. However, beef is also an ideal substrate for the growth of pathogenic microorganisms. Muscle foods, like beef, are defined as spoiled if organoleptic changes make them not acceptable to the consumer. These organoleptic changes could include, among others, changes in appearance (discoloration), the perception of off-odors, or even possible changes in taste. Obviously, such changes depend also on the type of species of microflora present in the meat, as well as the environment where the food is stored [3]. It has been widely accepted that visible organoleptic spoilage of meat is a result of decomposition and the formation of metabolites caused by the growth of microorganisms, which leads to the conclusion that growth to high numbers of bacteria is a precondition for meat spoilage [4]. Hence, the total viable count (TVC) of bacteria is considered as an important microbiological index for the quality and safety evaluation of meat as it “reflects” the level of freshness, contamination and spoilage. This index is useful to predict the shelf life of meat and distinguish meat spoilage during storage, as meat’s nutritious surface is favorable to the growth of a wide range of bacteria [5]. In general, a high TVC value in food indicates significant contamination by microorganisms and, potentially, a rapid spoilage situation.

The current practice to ensure the safety of meat still relies on regulatory inspection and sampling regimes. However, such procedure seems insufficient because it cannot satisfactorily guarantee consumer protection, since 100% inspection and sampling is technically, financially and logistically impossible. A variety of chemical, physical and microbiological-based methods have been explored for the detection and measurement of bacterial safety or spoilage in meat. These include enumeration methods based on microscopy and adenosine triphosphate (ATP) bioluminescence, as well as detection methods utilizing either immunological or nucleic acid-based procedures [6]. Nevertheless, these methods are time consuming, laborious, destructive and unsuitable for modern meat industrial processing and production technologies which prefer the adoption of techniques able to provide results in real time if possible [7]. During previous years, relevant analysis and screening methods had been carried out on meat utilizing expensive sensorial devices such as high-performance liquid chromatography (HPLC) and gas chromatography-mass spectrometry [8].

Alternative, non-expensive, rapid and noninvasive methods have been investigated for this purpose, not only to address consumers’ safety concerns but also to improve the level of efficiency in the meat supply chain in terms of profit and productivity. Such methods, involve various analytical lab instruments, like Fourier transform infrared spectroscopy (FTIR) [9], hyperspectral and multispectral imaging systems, Raman spectroscopy and electronic noses [10]. The detection approach of these sensorial devices is based on the concept that any produced metabolic activities for each sample of meat is considered as an individual “signature”, which practically includes essential information for the level of TVC [11]. With recent advances in imaging technology during the last decade, the spectral type of imaging (i.e., hyperspectral and multispectral) has been explored as a potential tool for monitoring the level of safety and quality of several agricultural products. This specific type of imaging concept, practically combines spectroscopy and conventional imaging approaches, and provides the user with spectral as well as spatial information of the examined product [12].

Multispectral imaging assisted with multivariate methods have been explored for the estimation of TVC in minced pork meat samples through the use of the VideometerLab (Videometer A/S, Hørsholm, Denmark) multispectral imaging (MSI) system [7], while an alternative imaging system, based on hyperspectral imaging (HSI) concept, has been investigated for the evaluation of tenderness in pork meat as well as for potential Escherichia coli contamination [13]. An HSI system has been also used to assess the TVC in pork samples during refrigerated storage, via acquired reflectance spectra extracted from sample images. Both partial least square regression (PLSR) and support vector machine regression (SVR) models for predicting TVC were built for comparative analysis [14]. A fusion of acquired spectral and texture features from a HSI system has been attempted as a way to improve the pH estimation in salted pork [15]. Adulteration of food has a long history and still is a common practice around the world. In the case of meat, it is linked with economic and safety implications, as it is rather challenging to detect specific ingredients and/or distinguish between species when evaluating meat visually. The detection of potential adulteration in minced beef has been explored through a HSI system [16], while a similar detection system utilizing multivariate techniques and machine learning algorithms has been used to distinguish between four processed beef burger patty categories, consisting of various ingredients and adulterants (pork, lamb, ostrich and textured vegetable protein) at selected levels [17].

One of the most important challenges with HSI/MSI systems, in terms of data analysis, is the extraction of useful information from the high dimensional hyperspectral data that contains redundant information. This has been achieved through the integration of modern analytical platforms with computational and chemometric techniques [18]. Dimensionality reduction techniques are generally applied to such cases in order to decrease the number of attributes needed for modelling. Such techniques not only speed up the data processing time but also contribute to a higher accuracy performance of the developed models. The current dimensionality reduction methodology mainly utilizes either the Principal Component Analysis (PCA) which is an unsupervised algorithm, or feature selection (FS) approaches, which are based on the concept of selecting specific wavelengths. These FS schemes have the advantage that the selected “original” attributes have a physical interpretation compared to the principal components found in PCA schemes. One main representative of these FS schemes includes partial least squares (PLSR) technique, a well-known regression approach, which has been also utilized as a method for dimensionality reduction based on the latent variables concept [19]. The choice of regression analysis method is an additional challenging problem for constructing a robust and universal model as well. Two well-known methods have been usually utilized; RLRS and support vector machine regression (SVR), a well-known nonlinear method that simplifies the regression problem by mapping the low dimensional nonlinear space into a high dimensional linear space based on kernel function [20].

The main objective of this paper is to associate spectral information acquired by a MSI system from aerobically stored beef fillet samples with the prediction of TVC of bacteria, an essential index for determining meat spoilage. The adopted approach provides an alternative methodology in predicting the TVC based on acquired MSI spectra. Although similar MSI-based approaches [21,22] utilize information only from the mean reflectance spectrum of the sample, the proposed methodology, extends this initial data source by considering additional information from the corresponding standard deviation of the pixels’ intensity per wavelength. Data analysis for TVC prediction is carried out separately for each of these first-order statistics (FOS) features (i.e., mean and standard deviation). For each FOS feature case, initially a number of well-known feature selection techniques have been employed to identify the most important attributes (i.e., wavelengths), and through a simple fusion scheme, based on majority voting, the most important wavelength variables are then chosen. These chosen wavelengths are then considered as inputs for a novel clustering-based asymmetric Gaussian fuzzy inference neural network (CAGFINN), which eventually predicts the TVC for that specific FOS feature case. The testing performances of CAGFINN models are compared against well-established machine learning methods, like support vector regressor (SVR), multilayer perceptron (MLP), PLSR, as well as an advanced wavelet neural network (WNN) scheme using a number of established evaluation metrics. Both individual predictions from CAGFINN models are then combined through a stacked-based ensemble nonlinear scheme which essentially calculates the final TVC prediction. Practically, the overall “idea” of the implemented methodology is to highlight the utilization of advanced learning-based models in the area of food microbiology, which could provide, potentially, an alternative approach for the detection of meat spoilage which currently relies mainly on the use of RLSR models.

## 2. Data Acquisition and Proposed Framework

### 2.1. Experiment and Data Acquisition

**Figure 1:**
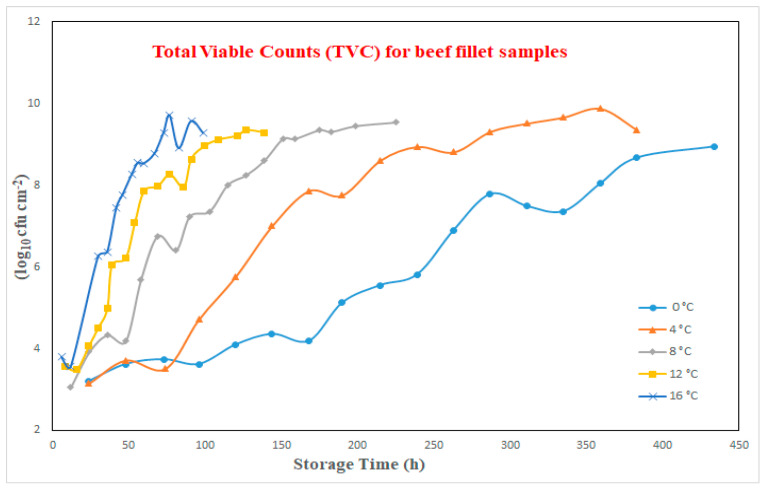
Growth curves of TVC at various temperatures.

The entire experiment and the associated data acquisition from the application of MSI system to meat samples, was accomplished at the Agricultural University of Athens, Greece. Details of the specific MSI-based experiment and the associated microbiological analysis for those beef samples used in the experiment can be found in [21]. Briefly, fresh beef filets were obtained from a meat market in Athens without performing any kind of preprocessing, like washing, removal of fat or connective tissue, before packaging. The acquired meat was divided into small portions of 50 g and packed aerobically in manually wrapped trays with air-permeable plastic film, ensuring that no direct contact of the film with the meat sample was occurred. These samples were then stored at the following temperatures 0, 4, 8, 12 and 16 °C in incubators for up to 434 h, subjected to storage temperature, until the effect spoilage was detected. At specific time-steps during storage, samples in duplicate were taken in order to be used for both microbiological and MSI-based analysis. Meat samples were analyzed at every 24 h intervals for the 0 °C and 4 °C cases, 10–12 h for the 8 °C cases, 6 h and 4–6 h for 12 °C and 16 °C cases, respectively. During microbiological analysis, the acquired growth data were log_10_ converted and a model, created by Baranyi and Roberts, was utilized to confirm various kinetic factors of microbial growth (maximum growth rate as well as lag phase length) for the TVC estimation. In this research study, in total, 84 meat samples were considered and the growth curves of TVC for fillet beef samples at those mentioned temperatures as a function of storage time are illustrated at Figure 1. An inspection at these graphs revealed that the TVC ranged from 3.058–9.885$\log_{10}\mathrm{cfu}\;{\mathrm{cm}}^{-2}$ and that the growth rate is increased faster as the storage temperature increases.

### 2.2. Multispectral Imaging Acquisition

Acquired multispectral images from each meat sample were obtained through the use of VideometerLab (Videometer A/S, Hørsholm, Denmark). The specific MSI system produces images in 18 distinct wavelengths, ranging from 405 to 970 nm. The wavelengths utilized in this MSI systems are: 405, 435, 450, 470, 505, 525, 570, 590, 630, 645, 660, 700, 850, 870, 890, 910, 940 and 970 nm. Meat samples were presented in Petri dishes and collected at the same time as microbiological analysis occurred [21]. The meat sample was set in a specially made (Ulbricht) sphere, which was coated with matte titanium paint and ensured a uniform reflection of the projected light so that a uniform light formed on the entire sphere. The digital camera on top of the sphere was a part of the receiving system, which gathered surface reflections and recorded the data with a standard monochrome CCD imaging sensor. Light-emitting diodes (LEDs) were set at the rim of the sphere and each wavelength of LEDs with narrowband spectral radiation distribution was evenly distributed across the entire edge. These characteristics undertake the best dynamic range and minimize the gloss-related effects, shadow effects and specular reflections. The result is a monochrome image with 32-bit floating point precision for each LED type, giving in the end, a multispectral 3D cube of dimensionality 1280 × 960 × 18 [23]. A necessary and important step in MSI system processing, after finishing image acquisition, is the image segmentation process which is to extract meaningful feature parts in the image, including the edges and regions. The objective of this process, using VideometerLab software (v.2.12.39), was to identify only the meat fillet as the Region of Interest (ROI) from the background or any other undesired regions such as the Petri dish as well as meat fat. To exclude the background from the images, a Canonical Discriminant Analysis (CDA) was considered, while a simple threshold used for segmentation [24].

**Figure 2:**
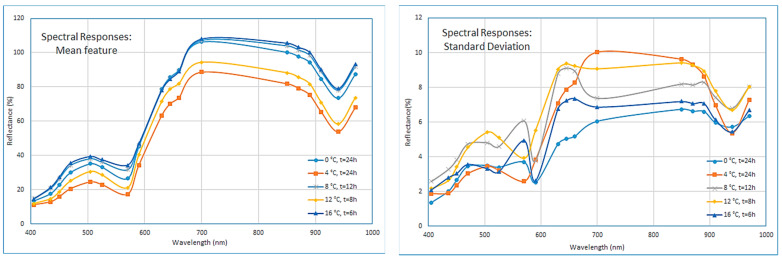
Selected spectral responses for “mean” and “sd” 1st order statistics features.

Following the segmentation process, for each image, and for each specific wavelength, the mean reflectance spectrum was obtained via the averaging the pixels’ intensity in the region of interest (ROI). In addition, for each wavelength, the standard deviation (sd) of the pixels’ intensity was also calculated [21]. The acquired mean reflectance values and their correlated standard deviations (totally 36 attributes) for each meat sample practically constitute the spectral signature for this specific sample. Figure 2 illustrate an example of mean and standard deviation reflectance spectra acquired from beef fillets samples, respectively. A close look on these selected “mean” spectra at Figure 2, lead to some interesting conclusions. Although these selected samples are associated with different temperatures, their “mean” spectra follow a common pattern with an increased trend in the reflectance’s magnitude especially for the visible part (400–700 nm), while their related reflectance values are decreased in the near infrared range. According to the literature, most of the spectral information used for meat discrimination is contained in the visible and near infrared region [25], therefore the proposed feature extraction process will also try to verify this specific scenario.

### 2.3. Integrated Analytical Framework

**Figure 3:**
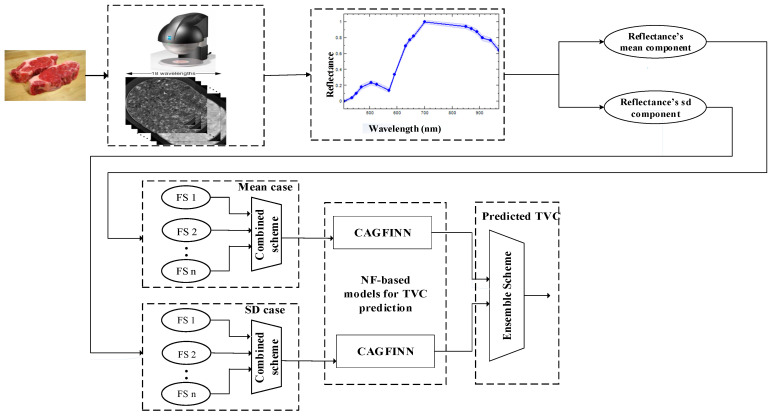
Proposed concept for data analysis.

Figure 3, illustrates the proposed analytic concept, following the imaging information acquisition from VideometerLab MSI system. The acquired spectral dataset includes two types of first-order statistics (FOS) features, the mean reflectance spectrum and the corresponding standard deviation of the pixels’ intensity per wavelength. Thus, each imaging data meat sample consisted of 18 wavelength attributes for each of these two FOS features, respectively. As illustrated from Figure 3, the analysis for these two FOS features has been implemented in parallel.

Initially, for each FOS feature case, seven well-known FS methods (Boruta, Recursive Feature Elimination, Genetic Algorithms and four regression-based schemes) have been investigated to identify their most important attributes (i.e., wavelengths). Unfortunately, there is not any FS algorithm that could provide an optimal performance in terms of dimensionality reduction. Thus, the main idea at this stage is to combine these individual selected wavelengths and choose the most important ones based on the majority voting criterion.

In parallel to this FS analysis, principal component analysis (PCA) has been also implemented for each FOS feature case, as PCA is a well-known dimensionality reduction scheme which usually appears in many food-related applications. One of the aims of this paper is to identify potential advantages of the proposed FS methodology over PCA. Two clustering-based neurofuzzy (NF) models have been employed to predict the TVC for each FOS feature case. The proposed NF model incorporates a “self-defined” clustering component in terms of self-determining the number of clusters, which eventually will equal to the number of fuzzy rules. In this paper, a number of well-known evaluation metrics have been applied to estimate NF’s performance. The overall TVC prediction is achieved through the use of a stacked ensemble scheme. The individual CAGFINN models are used as base-models in this proposed stacked scheme, while a nonlinear regression model acts as a meta-estimator. The use of a nonlinear regression model instead of the well-used linear regression, has been decided by the fact that such model can effectively combine the predictions of the base models in a nonlinear manner.

## 3. Features Extraction Methodology

### 3.1. A Principal Component Analysis Approach

In high-dimensional feature space cases, not-important attributes usually lead not only to unsatisfactory classification/regression accuracy, but also impose an added difficulty in finding potentially useful knowledge. This problem can be rectified through the use of Principal Component Analysis (PCA) technique [26]. In this study, the standard PCA algorithm has been applied on the multispectral information for both mean and sd feature components. As it is illustrated at Table 1, the first 5–6 principal components (PC) for each feature case can be utilized as input variables to the various regression models developed for this specific case study. PCA analysis is a standard and well-used dimensionality reduction approach in the area of food microbiology and these selected PCs, for both feature components, will be utilized as input vectors to the NF models at next stage in order to explore potential advantages of the proposed feature fusion scheme. The PCA analysis as well as the following feature selections methods were implemented through R (v.4.1.3).

### 3.2. A Fusion Approach of Feature Selection Algorithms

**Figure 4:**
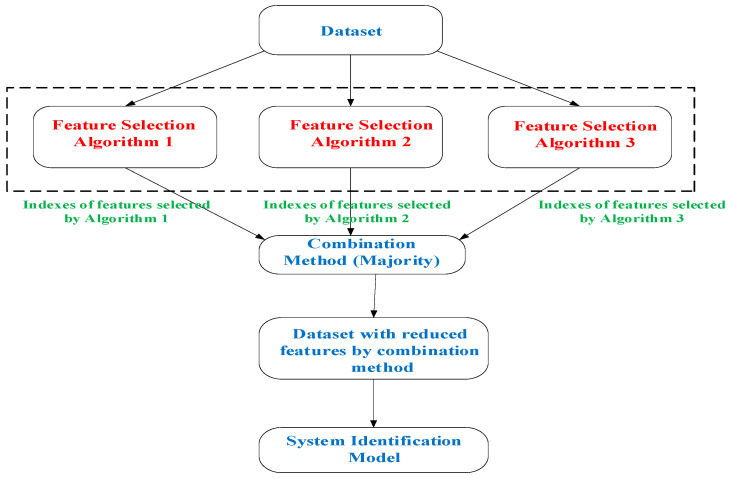
Proposed features fusion scheme.

As PCA is an unsupervised method, its extracted PCs that reflect the directions of highest variation are not necessarily associated to changes in the outcome variable (i.e., desired target). Additionally, such dimensionality reduction retains the issue of variables’ interpretation. The determination of finding which actual attributes are considered important is becoming a critical issue, and feature selection (FS) schemes provide an alternative solution to PCA. These schemes have been categorized into three groups, based on how they combine the selection algorithm and the model building: filter methods, wrapper methods and embedded methods. Many times, however, a randomly chosen FS method is selected with the expectation that it can perform the task adequately. In this paper, an alternative approach has been adopted in the context of FS: the combination or ensemble approach. The idea of combining multiple FS techniques is inspired from classifier ensembles [27]. The main concept behind this ensemble approach, is that we are not certain which specific wavelengths are the most important ones. In addition, many FS schemes have different criteria in the selection of their important variables. Under this proposed scenario, several well-known FS methods are implemented separately and their related important variables are extracted. The hypothesis in this paper is not only to assess the level of importance of these variables but also to check if common selected variables appear in these individual FS results. One simple way to implement this hypothesis, is a combination via the use of a voting scheme in order to choose the most relevant/important attributes. A general overview of the fusion approach is illustrated in Figure 4, where the “majority voting” concept has been chosen to fuse/select the final variables. For each FS technique, its associated list of variables is sorted based on their importance and the final output is a list which includes variables (i.e., wavelengths) that correspond to the most selective ones.

### 3.3. Feature Selection Techniques

Seven well-known FS methods have been chosen to contribute to the proposed fusion scheme shown in Figure 4. Initially, the Boruta algorithm and Recursive Feature Elimination (RFE), which both utilize random forest models for feature relevance estimation have been explored [28]. Genetic Algorithms (GA) attempt to computationally mimic the processes by which natural selection operates [29]. The remaining four schemes are practically regression-based methods; however, with variations in their operational “philosophy”. The Least Absolute Shrinkage and Selection Operator (LASSO) aims in minimization of the residual sum of squares while adding a constraint to the sum of the absolute values of the coefficients being less than a constant. A modified version of the stepwise (SW) technique, namely forward stepwise regression that utilizes the Akaike information (AIC) criterion [30] and the Relative Feature Importance (RFI) scheme [31] have been also considered. The final scheme is based on partial least squares regression (PLSR) which has been used widely in the area of Chemometrics [19]. The 18 distinct wavelengths obtained by the MSI system are shown in Table 2 with their associated coding (1 to 18). Similarly to the PCA case, FS techniques were applied separately to the “mean” and “sd” related features, respectively.

#### 3.3.1. Boruta Feature Selection Method

**Figure 5:**
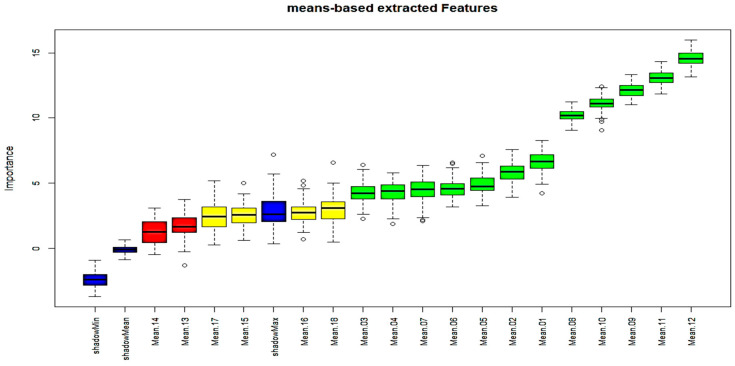
Important Variables based on Boruta scheme (mean case).

Boruta algorithm belongs to the wrapper family of FS techniques and is implemented around the random forest (RF) method. During its operation, the original dataset is extended by adding copies of original attributes (the so-called shadow attributes), as the main idea is to compare the importance of the real predictor variables with those shadow variables using statistical testing and several runs of RF algorithm. Figure 5 illustrates the results for “mean”-based wavebands revealing the related attributes’ importance. The columns in green are “confirmed” while the ones in red are not. During the decision process, some attributes shown with a yellow color are considered by the algorithm as “uncertain”. In the figure, the three blue bars are introduced by the algorithm itself and represent ShadowMax, ShadowMin and ShadowMean. Boruta’s advantage is that it clearly decides if a variable is important or not and helps to select variables that are statistically significant.

Based on these results, it seems that wavebands no. 12, 11, 9, 10, 8 and 1 are the first six most important attributes for the mean-based case, while, similarly, wavebands, no. 8, 12, 4, 6, 5 and 11 have been considered as important attributes for the sd-based case.

#### 3.3.2. Recursive Feature Elimination Selection Method

Recursive Feature Elimination (RFE) is probably the most used wrapper-based algorithm for selecting the most significant attributes. RFE utilizes a backward selection procedure in order to choose the ideal combination of input attributes. Initially, a model is created using all available attributes while their importance in the model is calculated. Then, the algorithm rank-orders the attributes and eliminates those with the least importance iteratively based on some evaluation metrics, like RMSE for regression.

**Figure 6:**
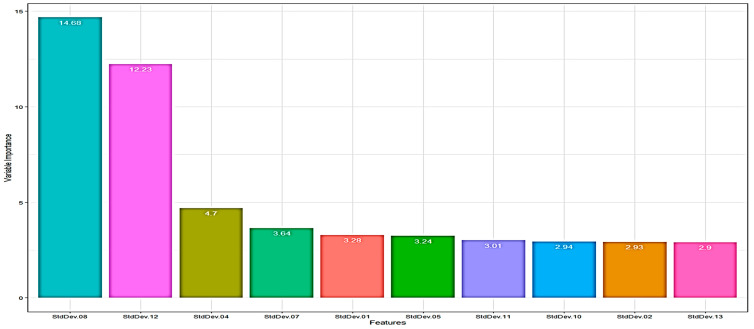
Important Variables based on RFE scheme (sd case).

After the removal of the least important attribute(s), the model is rebuilt and importance scores are computed again. Finally, the optimal attributes subset is then used to train the final model. In practice, RF algorithm is frequently used in this method due to use of some “post hoc” pruning of not essential attributes [32]. Figure 6 illustrates the outcome of RFE algorithm for RF based scheme for the “sd” spectral feature. For the “sd” case, the most important attributes are: “StdDev.08”, “StdDev.12”, “StdDev.04”, “StdDev.07”, “StdDev.01”, “StdDev.05” and “StdDev.11”, with the first two attributes to have a clear advantage over the remaining ones. Similarly, for the “mean” case, the most important attributes are: “Mean.12”, “Mean.11”, “Mean.09”, “Mean.10”, “Mean.08” and “Mean.01”.

#### 3.3.3. Genetic Algorithms (GA) Selection Method

Genetic Algorithms (GA) are inspired by Darwin’s theory of evolution from natural selection in the survival of the fittest. GA work with a set of candidate solutions (i.e., population) and produce sequential populations of alternate solutions that are represented by a chromosome. Chromosomes are evaluated for their quality, based to a given fitness function and are sorted accordingly. In this research, the gafs() R-based function, has been used to extract the most important attributes for both “mean” and “sd” cases. During the GA process, the appropriate crossover and mutation probabilities were set by the function to 0.8 and 0.1, respectively. In the final search, for the “mean” case, the following wavebands have been considered as important ones in that order: “Mean.01”, “Mean.09”, “Mean.12”, “Mean.11”, “Mean.07”, “Mean.08” and “Mean.05”, while for the “sd” case the related attributes were: “StdDev.04”, “StdDev.06”, “StdDev.07”, “StdDev.08”, “StdDev.12”, “StdDev.15”, “StdDev.16” and “StdDev.17”.

#### 3.3.4. Stepwise Selection Method

Stepwise regression is a regression-based model that has been used widely for FS processes. It can be implemented either by forward methods or backward methods, or by combing both methods. In this research, a hybrid scheme has been adopted, where the algorithm starts with forward selection and after each attribute (other than the first one) is selected to the model, a backward scheme is performed to check if any of those selected attributes could be eliminated without increasing significantly the Residual Sum of Squares (RSS). The orthogonal least squares algorithm has been used in this regression stepwise process. In our case study, for the “mean” case, the following wavebands have been considered as important ones: “Mean.12”, “Mean.07”, “Mean.02”, “Mean.01”, “Mean.09” and “Mean.05”, while for the “sd” case the final selected waveband attributes are: “StdDev.08”, “StdDev.06”, “StdDev.05”, “StdDev.04”, “StdDev.07”, “StdDev.02” and “StdDev.11”.

#### 3.3.5. LASSO Selection Method

**Figure 7:**
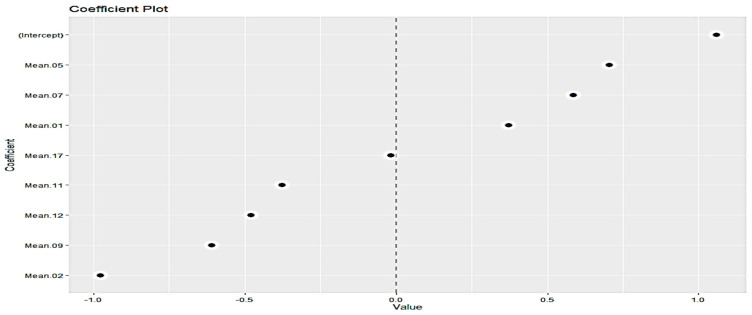
Important Variables based on Lasso scheme (mean case).

LASSO (Least Absolute Shrinkage and Selection Operator) regression is practically a modification of linear regression. The method tries to minimize the RSS but also adds a constraint to the sum of the absolute values of the coefficients being less than a constant. This modification allows LASSO to act as a FS method, because the shrinkage of the coefficients is such that some coefficients can be shrunk exactly to zero. The calculation of the amount of penalty/constraint, i.e., lambda (λ), by crossvalidation is a requirement, and for the “mean” and “sd” cases, the minimum lambda has been calculated as 0.0011 and 0.00066, respectively. Figure 7 illustrates the selection of appropriate attributes for “mean” case. Those attributes with either higher positive or lower negative values are considered as the most important ones. Thus, the selected wavebands based on Lasso scheme for the “mean” case are: “Mean.02”, “Mean.05”, “Mean.09”, “Mean.07”, “Mean.12”,“Mean.11” and “Mean.01”, while for the “sd” case: “StdDev.06”, “StdDev.05”, “StdDev.04”, “StdDev.08”, “StdDev.03” and “StdDev.07”.

#### 3.3.6. Relative Importance from Linear Regression Selection Method

In order to judge the relative importance of the input attributes in an approximation linear regression model, the magnitude and signs of the regression coefficients are normally considered. However, this approach is quite arbitrary, and sometimes unconvincing. Relative importance can be used to assess which attributes contributed to explaining the linear model’s R-squared value. Relative importance systems have been estimated using the Lindeman, Merenda and Gold (LMG) metric for correlated input relative importance [33]. The following attributes have been considered as important ones: “Mean.11”, “Mean.12”, “Mean.10”, “Mean.09”, “Mean.08”, “Mean.06”, “Mean.05”, “Mean.03” and “Mean.02”. The “sd” case, however, was proved to be more complex, as only few wavebands revealed a higher importance compared to the others. This proves the difficulty to model the “sd” case study via a linear regression approach. In this case, the most important attributes were: “StdDev.08”, “StdDev.12”, “StdDev.06”, “StdDev.04”, “StdDev.07” and “StdDev.05

#### 3.3.7. Partial Least Squares Regression (PLSR) Selection Method

**Figure 8:**
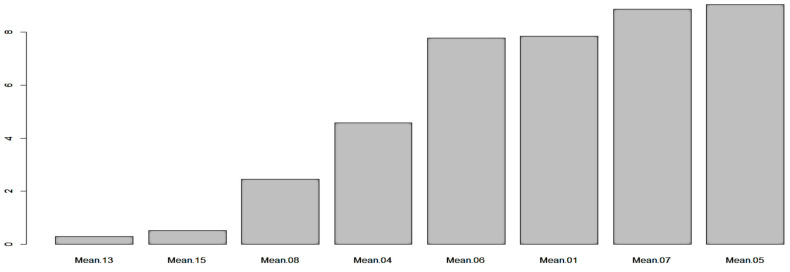
Important attributes based on PLSR scheme (mean case).

PLS regression method is probably the most used technique for FS in the area of food microbiology. It is an effective multivariate statistical method for analyzing the linear relationships between multiple independent and dependent variables. The idea of using PLSR in FS is based on filtering [34]. Wavelength bands are filtered based on the strength of the related regression coefficients. For the “mean” case study, the following attributes have been considered as important ones: “Mean.05”, “Mean.07”, “Mean.01”, “Mean.06”, “Mean.04”, “Mean.08”, “Mean.15” and “Mean.13”. Figure 8 illustrates the results from PLSR for “mean” case. The “sd” case, however, was proved again to be more complex, as more wavebands than the “mean” case revealed to have high in terms of magnitude coefficients. The most important features in this case were: “StdDev.04”, “StdDev.08”, “StdDev.05”, “StdDev.15”, “StdDev.10”, “StdDev.18”, “StdDev.07” and “StdDev.12”.

#### 3.3.8. Features Fusion

All selected features which have been extracted from those seven FS methodologies, are considered as inputs to the proposed feature fusion scheme. Table 3 illustrates the most important features (i.e., wavelengths) for each FS scheme for the “mean” case. For each FS method, chosen features are shown with their importance ranking. Only a few of the whole group of available wavelengths have been considered as important ones. Despite the different nature of these FS schemes, a number of specific wavelengths are common to the majority of these schemes. Based on ranking, as well as the importance level, the following wavelengths have been finally chosen for the “mean” case: “Mean.12”, “Mean.11”, “Mean.09”, “Mean.01”, “Mean.05”, “Mean.07”, “Mean.02” and “Mean.08”. These chosen wavelengths are also indicated in Table 3 via a red color.

A closer look to this table, reveals also an interesting outcome related to PLSR scheme, which has been extensively used as a FS method in many related applications in food microbiology. Specific wavelengths, not appeared in other methods, have been considered as important ones. This specific result verifies the choice of implementing a number of FS methods and their subsequent fusion. The selected wavelengths from this fusion scheme for the “mean” case, practically “verifies” the suggestion extracted from [25], as the most important information is mainly provided from wavelengths located at the visible part of the MSI system.

Similarly to the previous “mean” case, Table 4 illustrates the most important features (i.e., wavelengths) for each FS scheme for the “sd” case. Based on their importance ranking, the following wavelengths have been finally chosen for this case: “StdDev.08”, “StdDev.04”, “StdDev.05”, “StdDev.06”, “StdDev.12” and “StdDev.07”. Unlike to the previous case, fewer wavelengths are common to the majority of these FS schemes. Additionally, in some FS schemes, like GA and PLSR, a few wavelengths with a high wave number have been chosen. The diversity of selected wavelengths in the “mean” and “sd” cases, reveal also the importance of having multiple FOS features extracted from raw MSI images, which could potentially improve the overall TVC prediction.

## 4. Clustering-Based Asymmetric Neurofuzzy Architecture

**Figure 9:**
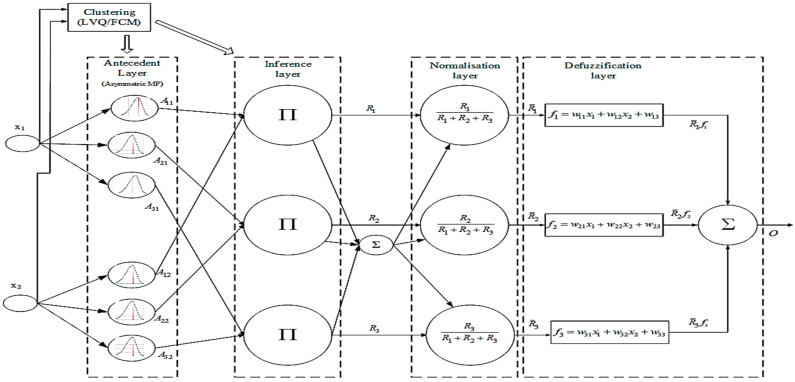
Illustration of CAGFINN system.

The proposed clustering-based asymmetric Gaussian fuzzy inference neural network (CAGFINN), shown in Figure 9, incorporates a “self-defined” clustering component in terms of self-determining the number of clusters which eventually will equal to the number of fuzzy rules. Similarly to other neuro-fuzzy models, CAGFINN follows the traditional TSK-based style, which has the advantage of modelling nonlinear processes with relatively fast training speed, due to the presence of linear units in its defuzzification part.

In the proposed CAGFINN structure the first three layers L1, L2 and L3 constitute the premise part (i.e., IF part) of a traditional fuzzy logic-based system, while layer L5 is associated to the consequent part (i.e., THEN part). Layer L4 is practically the normalization layer and is applied to the outputs from L3.

In this scheme, derived centers from the integrated clustering unit are used for the initialization for the centers of fuzzy membership functions (MFs). The proposed clustering scheme consists of two stages [35]. Initially, a fully supervised process which resembles with the well-known Learning Vector Quantization (LVQ) algorithm determines the number of clusters by dividing the data into these crisp groups and then calculates the initial coefficients of cluster centers. In the second stage, the classic fuzzy c-means (FCM) scheme is applied to optimize these coefficients of the cluster centers.

In the premise part of CAGFINN model, the number of produced clusters corresponds to the number of fuzzy rules. In addition, the spread parameters for each MF, $\sigma_{ij}$, are initialized based on the Equation (1),

(1) σij=∑k=1nuik(xkj−cij)2/∑k=1nuik12

where elements in matrix U correspond to the MFs of input $x_{k}$ from the ith cluster, as they were calculated by the FCM scheme used at the second stage of proposed clustering initialization process. Thus, fuzzy rules can be formulated by the following equations, based on the adopted configuration:

(2) $$\begin{array}{l} \mathrm{IF}\;(x_{1}\;\mathrm{is}\;U_{1}^{i}\;\mathrm{AND}\ldots \mathrm{AND}\;x_{q}\;\mathrm{is}\;U_{q}^{i})\;\mathrm{THEN}\;y=\sum_{j=1}^{c}f_{j}R_{j,}\\ \mathrm{where},\;f_{j}=w_{j1}x_{1}+\ldots +w_{jn}x_{n}+w_{j(n+1)} \end{array}$$

where U are the sets derived from the c-partition of training data X and $R_{c}$ are the fuzzy normalized rules. The proposed configuration of CAGFINN schemes is explained below layer by layer:

**Layer 1:** Nodes at this stage simply forward input variables $x_{1},\;x_{2},\;\ldots ,\;x_{n}$ to L2.
**Layer 2:** This is premise part in a fuzzy IF–THEN structure, where utilizes an asymmetric Gaussian MF with the following form:
  (3) $$\begin{array}{l} A_{ij}=\exp\left(-\frac{1}{2}{\left(\frac{x_{i}-c_{ij}}{\sigma_{ij}^{left}}\right)}^{2}\right)U\left(x_{i};-\infty ,c_{ij}\right)+\\ \;\;\;\;\;\exp\left(-\frac{1}{2}{\left(\frac{x_{i}-c_{ij}}{\sigma_{ij}^{right}}\right)}^{2}\right)U\left(x_{i};c_{ij},\infty \right)\\ where\;U\left(x_{i};a,b\right)=\left\{\begin{array}{l} 1\;\;if\;a\leq x_{i}<b\\ 0\;\;otherwise \end{array}\right. \end{array}$$

The MF in Equation (3) includes two types of spreads, namely $\sigma_{ij}^{left}$ and $\sigma_{ij}^{right}$, respectively, which transform the classic Gaussian MF to a nonsymmetric output form. Moreover, this approach utilizes a “pseudo” function that requires two spreads—left and right—and a common center parameter. Following the FCM clustering stage, an initial value for spread ($\sigma_{ij}^{\mathrm{init}}$) has been calculated. As CAGFINN utilizes two spreads, one located at the left of the initial center parameter and one at the right, both spreads are initialized as $\sigma_{ij}^{\mathrm{init}}/2=\sigma_{ij}^{left}=\sigma_{ij}^{right}$. Thus, during the first iteration of the training process, the total spread of any asymmetric MF has been equaled to $\sigma_{ij}^{total}=\sigma_{ij}^{left}+\sigma_{ij}^{right}$. Upon the arrival of any input variable from L_1_, its position (left/right) against the specific center parameter for each MF needs to be recorded via a specific MF index allocated for each MF. This index is then used in the backward learning phase to update that particular spread parameter. The value of this index is then updated accordingly to any new input arrival from L_1_. During forward training phase, $\sigma_{ij}^{total}$ is used as the spread used in the Gaussian function which has the specific form:

(4) $$A_{ij}=\exp\left(-\frac{{\left(x_{i}-c_{ij}\right)}^{2}}{2b_{ij}^{2}}\right)$$

where $b_{ij}=\sigma_{ij}^{total}$, i represent the number of MF/rules, while j denotes the specific input variable. During the backward learning phase, a new spread $\sigma_{ij}^{new}$ value is calculated via the gradient descent (GD) learning method. Based on the information stored at that specific MF index, either the left or right spread is updated as $\sigma_{ij}^{left\;\mathrm{or}\;\mathrm{right}}=\sigma_{ij}^{new}/2$. For the next iteration step, in the forward training phase, the spread parameter will be equal again as $\sigma_{ij}^{total}=\sigma_{ij}^{left}+\sigma_{ij}^{right}$, incorporating, however, the relative adjustment of one of its components.

**Layer 3:** This layer represents the “classic” rules layer. The multiplication has been used as a fuzzy AND operation, thus output has the following form:
  (5) $$R_{i}=\prod_{j}^{n}A_{ji}(x_{j})$$

In this proposed architecture, the number of rules is the same as the number of clusters.

**Layer 4:** At this normalization layer, each normalized rule is calculated by:
  (6) $${\bar{R}}_{i}=\frac{R_{i}}{\sum_{j=1}^{c}R_{j}}$$
**Layer 5:** Finally, this is the consequent stage of the CAGFINN scheme which has the following form:
  (7) $$O=\sum_{j=1}^{c}f_{j}{\bar{R}}_{j}$$
  where $f_{j}=w_{j1}x_{1}+\ldots +w_{jn}x_{n}+w_{j(n+1)}$ illustrates the “consequent linear parameters” of the TSK-based scheme. Inspired by ANFIS’s hybrid learning algorithm, similar analysis has been performed for the case of CAGFINN scheme. Τraining process includes the use of recursive least squares method (RLS) for the consequent parameters, while the gradient descent (GD) algorithm has been employed for the premise part [36]. During, the backward “training” phase, the error signals are calculated from the output layer backward to the premise (i.e., membership) layers, and parameters at both defuzzification and fuzzification sections are fine-tuned. All modelling schemes for the TVC prediction have been implemented in MATLAB (ver. R2019b, Mathworks.com (accessed on 1 October 2023)).

## 5. Predictive Models for the Estimation of Total Viable Counts in Meat Samples

The main objective in this paper is the efficient estimation of TVC on beef samples based only on data acquired by an MSI system. No additional information, such as the specific time-steps at which beef samples were analyzed during storage, or the classification groups (fresh, semifresh or spoiled) of the acquired samples was utilized. The rationale of using only spectral information was justified by the fact, that sometimes such additional information may not be instantly available. The main concept of the proposed framework, shown at Figure 3, is the TVC prediction not via a single regression model, but rather through an ensemble scheme, where the predictions of its individual FOS-based feature components through the aid of a metalearning algorithm, are combined in order to produce the final outcome. Two regression models, based on the proposed clustering-based neurofuzzy regression model (CAGFINN), have been trained and evaluated separately for the “mean” and “sd” cases, respectively. However, as we are interested, initially, to assess the performance of CAGFINN scheme acting as TVC prediction model, obtained results are compared with those obtained by MLP neural networks, SVM, PLSR schemes and wavelet neural networks (WNN). Although, MLP and PLSR schemes have been already applied in this domain [37], the comparison is also extended with the addition of SVM as well as an advanced WNN model [38]. The main reason for using this specific WNN model, is related to the fact that this structure includes an additional linear-coefficients-weights layer that resembles the TSK defuzzification scheme found in CAGFINN. The dataset, consisted of 84 meat samples, include the selected input variables (i.e., wavelengths) chosen by the feature selection fusion scheme, and the associated TVC prediction obtained by microbiological analysis. In this research study, two distinct procedures have been considered for the training/testing stages. In the first procedure, as the number of observations/samples is small, the separation of the dataset into training and testing subsets (hold-out method) was considered that it would further reduce the number of data and would result in insufficient training of the network. Therefore, in order to improve the robustness of identification process, the leave-1-out cross validation (LOOCV) technique was employed to evaluate the performance of all developed models for both “mean” and “sd” feature cases. In order to investigate further the capabilities of the implemented models for the TVC prediction, a second experiment was also carried out, where the initial dataset was divided into a training subset with approx. 90% of the data and a testing subset with the remaining 10% (i.e., eight samples). The performances of developed regression models for the prediction of TVC, for both “mean” and “sd” cases, are evaluated through a number of well-established metrics. Initially the Bias factor (B_f_) and accuracy factor (A_f_) performance indicators, which have been extensively used in food microbiology, have been applied [39]. The bias factor measures the bias of the data, while the accuracy factor measures of the scatter of the data. These indicators are shown in the following equations:

(8) $$B_{f}=10^{\left(\sum \log\left({\overset{⌢}{y}}_{i}/y_{i}\right)/n\right)}$$

and

(9) $$A_{f}=10^{\left(\sum \left|\log\left({\overset{⌢}{y}}_{i}/y_{i}\right)\right|/n\right)}$$

where $\overset{⌢}{y}$, y represent the predicted and desired responses, while n is the number of observations. The usual measures of goodness-of-fit for model comparison in food microbiology is performed, in addition to B_f_ and A_f_ with the squared correlation coefficient (R^2^), known also as coefficient of determination. In our case, it can be interpreted as the proportion of the variance in the predicted TVC response from the actual/objective TVC response. The higher the value (0 ≤ R^2^ ≤ 1), the better is the prediction by the model. The coefficient of determination and its equivalent correlation coefficient (R) is also one of well-known metrics used for determining the effect size. Effect size is a statistical concept that measures the strength of the relationship between two variables on a numeric scale and is used mainly in health-related domains, like psychology. Alternatively, the effect size, as a way to compare two variables, is also calculated as the difference between the two variables’ means. Two similar metrics of this type are also considered in this paper for the evaluation of the implemented regression models [40]. The first is the standardized mean difference (theta) scheme, which is shown in the following equation:

(10) $$theta=\frac{\bar{y}-{\bar{y}}_{calc}}{\sigma_{y}}$$

where $\bar{y}$, ${\bar{y}}_{calc}$ represent the means of the desired and predicted response, respectively, while $\sigma_{y}$, is the standard deviation of the desired response. A slightly different approach is the Cohen’s d effect size metric which is defined as difference between the two means divided by the pooled standard deviation from the data and is defined by the following equation.

(11) $$cohen’s\;d=\frac{\bar{y}-{\bar{y}}_{calc}}{\sigma_{pooled}}=\frac{\bar{y}-{\bar{y}}_{calc}}{\sqrt{\frac{(n_{y}\;-\;1)\sigma_{y}^{2}\;+\;(n_{ycalc}\;-\;1)\sigma_{ycalc}^{2}}{n_{y}\;+\;n_{ycalc}}}}$$

Cohen’s suggestion that effect sizes can be categorized as small, medium or large is associated with the amount of overlap of the two involved variables.

However, R^2^ is considered as a suitable criterion for model comparison on the assumption that the error is normally distributed and not dependent on the mean value. In fact, the distribution of the error is not clearly known in the case of microbial/bacteria growth, so this index must be used with caution, particularly in nonlinear-based regression models and hence additional evaluation metrics must be employed for model comparison [41]. Inspired by the regularly used metrics in regression problems, the mean absolute percentage error (MAPE), the root mean squared error (RMSE), the standard error of prediction (SEP), the absolute percentage error (APE) and the mean absolute error (MAE) metrics have been also considered as evaluation measurements for our case study [35].

Finally, three additional chemometric indicators, which are used extensively in spectroscopic applications, namely residual prediction deviation (RPD), range error ratio (RER) and the ratio of performance to interquartile distance (RPIQ), were utilized in this paper. The RPD is calculated as the ratio of the standard deviation of the desired variable to the RMSE. Higher values for the RPD suggest increasingly accurate models and a model with RPD value above three is generally considered as an excellent model in terms of reliability [42]. The RPD value although is considered as an important criterion, it assumes a normal distribution of the desired/observed values since it includes the standard deviation. An alternative metric is the Ratio of Performance to InterQuartile distance (RPIQ), which is defined as interquartile range of the observed values divided by the RMSE. RPIQ is based on quartiles, which better represent the spread of the dataset. The quartiles are milestones in the dataset range: Q_1_ is the value below which we can find 25% of the samples while Q_3_ is the value below which we find 75% of the samples. The RPIQ formula is shown as follows:

(12) $$RPIQ=\frac{IQ}{RMSE}=\frac{Q_{3}-Q_{1}}{\sqrt{\frac{1}{n}\sum {\left(y_{i}-{\hat{y}}_{i}\right)}^{2}}}$$

The RPIQ takes both the prediction error and the variation of observed values into account, providing thus a metric of model validity that is more objective than the RMSE. A larger RPIQ value indicates improved model performance. Finally, the range error ratio (RER) is equal to the range in the observed/desired values (i.e., the maximum value minus the minimum value) divided by the RMSE. A value of RER above 10 is roughly an indicator of a model with good predictive ability [43].

### 5.1. “Mean” Feature Case Study

All regression models, including CAGFINN, have been implemented utilizing the same input vector that includes eight input nodes (i.e., the selected eight “mean” features from the proposed fusion scheme). In the proposed neurofuzzy (NF) scheme, 12 clusters have been created, using the clustering preprocessing stage for the LOOCV case, while 10 clusters for the 2nd experiment with the reduced training dataset case.

**Figure 10:**
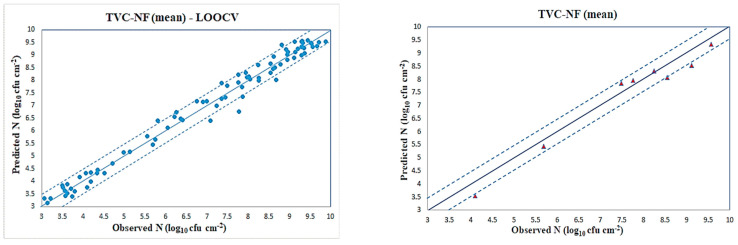
CAGFINN models for TVC prediction (“mean” case).

The number of fuzzy rules is the same as the number of MIs and is independent from the number of input variables, creating thus a novel “multidimensional inspired” rule layer, in contrast to in contrast to traditional ANFIS architecture [44]. The hybrid learning algorithm, resulted in a fast-training process, which was concluded in less than 500 epochs, much faster from the equivalent time used to train the MLP neural network. The scatter plot of predicted (via CAGFINN) versus observed total viable counts for the LOOCV case is illustrated in Figure 10, and shows a very good distribution around the line of equity (y = x), with the vast majority of the data included within the ±0.5 log area. A few samples, however, are located in the borders of the designated ±0.5 log area, while five samples, namely “49A1”, “51A1”, “55A1”, “50A3” and “19A7” are clearly located outside that zone. It is interesting to investigate the characteristics of these five “outliers” samples. The “49A1”, “51A1” and “55A1”samples were stored at 0 °C and collected after 239 h, 263 h and 383 h time of storage, respectively. The “50A3” sample was stored at 4 °C and collected after 263 h time of storage and finally the “19A7” corresponds to a beef sample stored at 12 °C and collected after 54 h time of storage. Although this “outliers” set includes samples from different storage temperatures, the performance of NF model from this plot reveals a rather “nonlinear” trend as many samples are distributed above and below from the line of equity. Practically, this plot illustrates the nonlinear characteristics of the TVC prediction for the “mean” case.

The performance of NF model, using the chosen wavelengths as input variables, is revealed with more details though the application of the evaluation statistical metrics, presented in Table 5. The bias and accuracy factors revealed a rather perfect fit, with values 1.003 and 1.035, respectively. B_f_ is used to determine whether the model over or under predicts the level of bacterial growth. A B_f_ greater than 1.0 indicates that a model is overestimated. Equally, a B_f_ less than 1.0 generally indicates that a model is underestimated (i.e., observed responses are larger than predicted values) [44]. Perfect agreement between predictions and observations would lead to Bf of 1. Regarding the appropriate values of the A_f_, a value of one indicates that there is perfect agreement between all the predicted and measured values. All remaining evaluation results reveal a rather excellent regression performance for the proposed NF model for the LOOCV case. The R-squared result has been calculated as 0.982, which is considered as an accepted value to evaluate the goodness-of-fit for this particular model. Cohen’s d test is almost zero, which reveals a strong overlapping between predicted and observed responses. The obtained high RPD shows that this model can definitely be used for such prediction tasks. In parallel, an additional CAGFINN model has been implemented using, however, as input variables the PCs from the PCA scheme. The aim was to compare the concept of the proposed fusion FS vs. the traditional PCA approach. In this PCA/NF version, 8 clusters have been created, using the clustering preprocessing stage for both the LOOCV case as well as the second experiment with the reduced training dataset case. Although, this PCA/NF-based model contains fewer input variables than the equivalent FS-based model, results were slightly inferior. This comparison proved the validity of the hypothesis to utilize selected wavelengths as input variables in order to have a greater level of understanding of the model.

In addition to CAGFINN model, WNN, MLP, SVM and PLSR models have been also developed to predict TVC. The concept of using wavelets in neural networks is well known and is summarized by the replacement of the Gaussian function, normally found in RBF networks, with an orthonormal scaling functions; however, in the proposed advanced WNN-LCW architecture, the connection weights between the hidden layer neurons and output neurons are replaced by a local linear model, similar to the output TSK-layer appeared in ANFIS neuro-fuzzy system [38]. From this point of view, there is a level of similarity between CAGFINN and WNN-LCW models, and results also reveal the importance of utilizing such a “defuzzification” layer in learning-based models. In the proposed WNN-LCW, 20 wavelet Morlet functions have been used and the evaluation performance of the developed WNN was comparable with the PCA/NF model. For the case of SVM, the criterion for building the optimal model was defined by the evaluation of SEP index. The C ranges were chosen in between [1 and 1000] and γ value ranged from [0.05 to 1] for training the ε−SVR. The epsilon tolerance value was set to be 0.001. Penalty coefficient C was set at 120, while γ parameter at 0.25. An MLP network has also been implemented, utilizing two hidden layers (with 16 and 8 nodes, respectively). Both performances from MLP and SVM models, although inferior compared to previous models, reveal nevertheless a robust performance, taking into consideration that such models have been already used in the area of food microbiology. Finally, a partial least squares regression (PLSR) scheme has been applied to the same dataset. Although SVM and MLP performances could be characterized as comparable, obtained PLSR results are worse than those obtained by other machine learning schemes. It is well known that in modelling of real processes, linear PLSR has some difficulties in its practical applications since most real problems are inherently nonlinear and dynamic [45]. In fact, a close inspection at results in Table 5, reveals that RPD index is less than 3, revealing that such a model cannot be considered as acceptable.

Similarly, all developed models have been also utilized in a second simulation study, in order to assess their competence to be trained with a dataset with a reduced number of samples. In this scenario, 76 samples were used for training purposes, while the remaining 8 samples were reserved for the evaluation. The plot of predicted (via CAGFINN) versus observed total viable counts for this second case is illustrated in Figure 10, while the performance of the all developed models to predict TVCs in beef samples in terms of statistical indices is presented in Table 6. Results from this table indicate again the superiority of implemented regression models over PLSR, even though they are inferior to those illustrated in Table 5. In this case, all B_f_ indices are less than 1, indicating the underestimation nature of all models. However, a closer comparison of performances in these two tables, reveals a problem with the limited number of samples for training. All evaluation metrics are much worse in this second case, and this reflects an open problem in learning-based systems, i.e., the need to have as large as possible training datasets.

### 5.2. “SD” Feature Case Study

For this case, the input vector for all models consisted of the chosen six “sd” features from the proposed fusion scheme. In the proposed NF model, 14 clusters were created, using the clustering preprocessing stage for the LOOCV case, while 12 clusters were created for the second experiment. The increased number of clusters/rules indicates indirectly the different and rather more complex characteristics of the “sd” feature compared to the “mean” case. In this case, the structure for WNN-LCW consisted of 16 wavelet Morlet functions, while the MLP retained the same internal structure as in the previous “mean” case.

**Figure 11:**
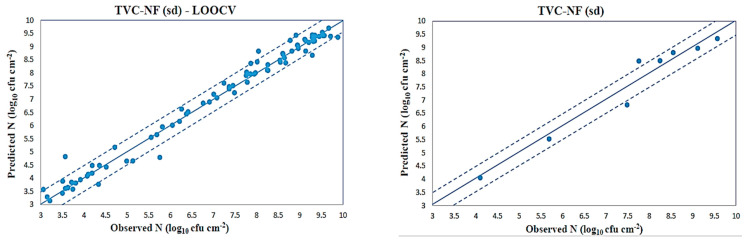
CAGFINN prediction models for TVC (“sd” case).

The plots of predicted (via CAGFINN) versus observed total viable counts for both simulation studies are illustrated in Figure 11, and show a good distribution around the line of equity (y = x), with the vast majority of the data included within the ±0.5 log area. A few samples, however, are located in the borders of the designated ±0.5 log area, while four samples, namely “36A3”, “4A9”, “54A1” and “26A9”, are clearly located outside that zone. The “54A1” sample was stored at 0 °C and collected after 359 h time of storage, while “36A3” to a sample stored at 4 °C and collected after 120 h time of storage. Finally, “4A9” and “26A9” samples were stored at 16 °C and collected after 12 h and 73 h time of storage, respectively. This plot, however, in contrast to the previous “mean” case, reveals a rather “linear” trend as many samples are located on the line of equity. Such difference in the trends shown at Figure 10 and Figure 11, illustrate the different characteristics embedded in both “mean” and “sd” cases. The performances of all the developed models to predict TVCs in terms of statistical indices for the LOOCV case are presented in Table 7.

Looking at these results, shown in Table 7, a difference in modelling the TVC using the “sd” feature is more than evident. Although NF model achieved a superior performance in all metrics, it is interesting to explore the performance of the WNN model. Both NF and WNN models share a common defuzzification component, which is responsible for obtaining superior performance in single output nonlinear regression problems. The R-squared in this case is 0.981 for the NF scheme compared 0.654 for the PLSR, revealing the deficiency of this particular model in modelling nonlinear problems.

Similarly, all developed models have been also utilized in a second simulation study, in order to assess their competence to be trained with a dataset with a reduced number of samples. The plot of predicted (via CAGFINN) versus observed total viable counts for this second case is illustrated in Figure 11, while the performance of the all developed models to predict TVCs in beef samples in terms of statistical indices is presented in Table 8.

Results in this case, verify again the superiority of the proposed models over PLSR scheme. The obtained RPD and R-squared results for the PLSR model shows how inferior is such a model which currently is used extensively in the area of food microbiology. On the other hand, the proposed CAGFINN clearly outperforms both MLP and SVM schemes, which are very popular in the regression analysis, although their overall performance can be considered as acceptable for modelling a nonlinear process. In fact, RPD values for MLP and SVM in this second experiment were above 3, while also the equivalent R-squared metric is acceptable for both models.

### 5.3. Ensemble System

In general, ensembling is a technique of combining two or more algorithms/models of similar or dissimilar types, called base learners. The main idea is to make a more robust system which incorporates the predictions from all the base learners [46]. A single algorithm/model may not be able to provide the perfect prediction for a given dataset. Each machine learning algorithm has its own limitations and thus producing a model with high accuracy is challenging. However, a simple way to implement such combination of models is by aggregating their outputs. Such aggregation can be realized by using different techniques, sometimes referred to as meta-algorithms.

Although the “mean” feature had been considered as the main information provider for the case of multispectral imaging analysis [21], in our specific research case study, both “mean” and “sd” individual FOS features have been analyzed separately. For each case, an advanced NF model has been employed to predict the required TVC in beef samples.

Even though a satisfactory TVC prediction was achieved for each case, the question is how we can combine these individual TVC predictions to obtain an even better result, if possible. The first “combination” approach is the well-known averaging scheme. It is defined by taking the average of predictions from models in case of regression problem. This approach was applied to CAGFINN’s predictions for both “mean” and “sd” cases and results are summarized at Table 9 and Table 10. Obviously, an improvement in overall TVC prediction has been achieved, compared to the individual “mean” TVC prediction. For example, the RPD, SEP or the R-squared values reveal a clear robustness of the averaging final model for TVC prediction, while B_f_ is almost perfect.

However, the main aim in this section is the application of a stacking ensemble scheme to this specific problem. Stacking is another ensemble scheme which involves training of a new learning algorithm/model to combine the predictions of several base learners. First, the base learners are trained using the available training data and then a combiner or meta-algorithm/model, called the super learner, is trained to make a final prediction based on the predictions of the base learners [47]. In contrast to bagging and boosting ensemble schemes, stacking is designed to “combine” a diverse group of strong learners. In our case, the two CAGFINN models (“mean” and “sd” case) were used for TVC prediction were used as base learners. This metamodel has been trained using the TVC (training) predictions made by base models, rather than the data used to train the base models. These predictions along with the desired TVC outputs, provide the input and output pairs of the training dataset used to train the metamodel.

In this paper, two algorithms were used as potential “metamodels”, the PLSR and a nonlinear regression using the nonlinear iterative partial least squares (NIPALS) algorithm. All ensemble schemes were developed using XLSTAT v.2019.2 software. For the case of nonlinear “metamodel”, the following fourth order model being constructed.

$$\begin{array}{l} Y=1.37-51.38*X_{mean}+50.65\;*X_{sd}+17.71*{\left(X_{mean}\right)}^{2}-16.98*{\left(X_{sd}\right)}^{2}\\ -2.87*{\left(X_{mean}\right)}^{3}+2.73*{\left(X_{sd}\right)}^{3}+0.22*{\left(X_{mean}\right)}^{4}-0.21*{\left(X_{sd}\right)}^{4} \end{array}$$

**Figure 12:**
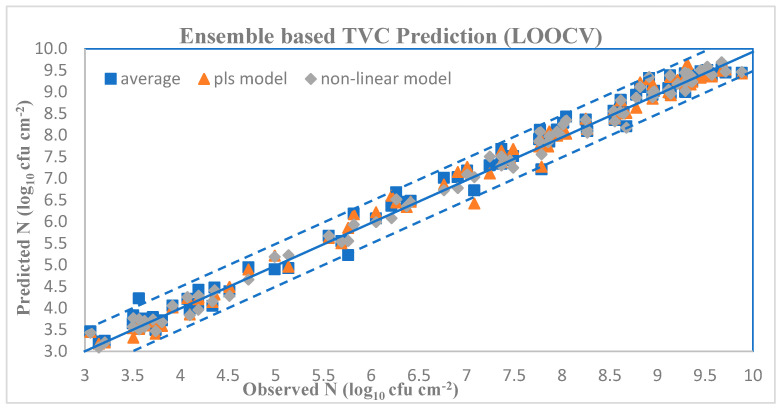
Ensemble scheme TVC predictions (LOOCV).

**Figure 13:**
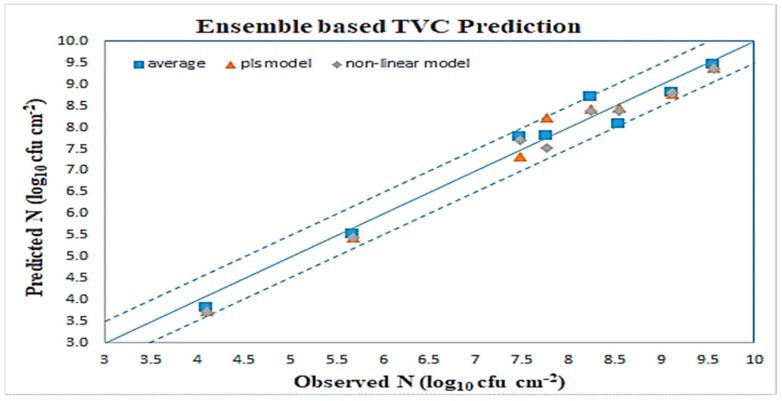
Ensemble scheme TVC predictions (reduced-dataset).

Related results are shown in Table 9 and Table 10. The scatter plot for all ensemble schemes in the LOOCV case is illustrated in Figure 12, and shows a very good distribution around the line of equity (y = x), with the vast majority of the data included within the ±0.5 log area. The performance of the proposed nonlinear metamodel is superior compared to the one obtained via the PLSR metamodel. Evaluation metrics for both PLSR and nonregression models, shown at Table 9 and Table 10, reveal a clear advantage over the standard averaging combination. Looking at Figure 12, the majority of cases in nonlinear scheme are closer to the line of equity, while a few cases from averaging and RLSR schemes are close to border lines or even outside. Similarly, for the reduced dataset simulation case, the equivalent scatter plot is shown at Figure 13. Results also in this case verify the validity of the hypothesis of using a metamodel scheme to predict TVC in beef samples.

Although, in this specific case study, only two base models have been used and thus the associated complexity is not considered as too high, it was interesting to investigate the performance of a metamodel that develops a nonlinear mapping from the predictions of bottom layer models to the outcome.

## 6. Conclusions

In conclusion, this simulation study demonstrated the effectiveness of the proposed machine learning based framework that could be considered as an alternative technique for accurate prediction of TVC of microorganisms in meat samples. The first part of this framework includes the application of a number of FS methods which select specific wavelengths and a simple fusion scheme that eventually choose the most important of them. As two different sources of information (“mean” and “sd”) are extracted from multispectral images, the second part included the concept of an ensemble stacking scheme, which utilized two advanced neurofuzzy (CAGFINN) regression models acting as base models and one nonlinear regression algorithm acting as a metamodel. The performance of CAGFINN models was evaluated successfully through a number of established metrics and compared against algorithms which are currently utilized in the area of food microbiology, like MLP, SVM and PLSR as well as an advanced wavelet neural network model. The nonlinear metamodel in the proposed ensemble scheme provided a robust regression performance, which eventually verified the effectiveness of the proposed framework. Future work will concentrate in modifying CAGFINN’s architecture from a classic MISO to MIMO configuration. Obviously, that also requires a related reform of the learning algorithm to address such changes, especially to the issue of current TSK defuzzification scheme. Such modification will be very useful in this specific application, as in addition to TVC prediction, a MIMO model could also predict the status/class of the meat sample, providing thus a more complete analysis towards the detection of meat spoilage.

## Figures and Tables

**Table 1:** PCA analysis for “mean” and “sd” feature cases.

PCs	PCA-Mean			PCA-sd		
	Eigenvalue	Prop. %	Cum. Prop. %	Eigenvalue	Prop. %	Cum. Prop. %
1	726.577	77.043	77.043	46.397	81.975	81.975
2	126.239	13.386	90.429	3.735	6.600	88.574
3	72.162	7.652	98.081	3.154	5.573	94.147
4	14.212	1.507	99.587	2.020	3.568	97.715
5	2.037	0.216	99.803	0.579	1.023	98.739
6	0.657	0.070	99.873	0.274	0.484	99.223

**Table 2:** List of wavebands (nm).

No:	1	2	3	4	5	6	7	8	9	10	11	12	13	14	15	16	17	18
Wavebands (nm)	405	435	450	470	505	525	570	590	630	645	660	700	850	870	890	910	940	970

**Table 3:** Proposed Fusion for “mean” case.

Method\Bands No.	1	2	3	4	5	6	7	8	9	10	11	12	13	14	15	16	17	18
Boruta	6th	7th			8th			5th	3rd	4th	2nd	1st						
RFE	6th	9th					7th	5th	3rd	4th	2nd	1st			8th			
Stepwise	4th	3rd			6th		2nd		5th			1st						
Lasso	7th	1st			2nd		4th		3rd		6th	5th						
GA	1st				7th		5th	6th	2nd		4th	3rd						
Relative Importance		9th	8th		7th	6th		5th	4th	3rd	1st	2nd						
PLSR	3rd			5th	1st	4th	2nd	6th					8th		7th			

**Table 4:** Proposed Fusion for “sd” case.

Method\Bands No.	1	2	3	4	5	6	7	8	9	10	11	12	13	14	15	16	17	18
Boruta		7th		3rd	5th	4th		1st			6th	2nd						
RFE	5th			3rd	6th		4th	1st			7th	2nd						
Stepwise		6th		4th	3rd	2nd	5th	1st			7th							
Lasso			5th	3rd	2nd	1st	6th	4th										
GA				1st		2nd	3rd	4th				5th			6th	7th	8th	
Relative Importance			7th	4th	6th	3rd	5th	1st				2nd	8th					
PLSR				1st	3rd		7th	2nd		5th		8th			4th			6th

**Table 5:** Performance of developed models for TVC (mean—LOOCV case).

Statistical Index “Mean” (LOOCV)	PCA/NF	FS/NF	WNN	MLP	SVM	PLSR
*Root mean squared error (RMSE)*	0.3492	0.2863	0.3477	0.3898	0.4038	0.7541
*Mean absolute percentage error (MAPE %)*	3.8738	3.4972	4.2597	4.9223	4.8697	9.3105
*Standard error of prediction (SEP %)*	4.9821	4.0857	4.9619	5.5627	5.7624	10.759
*APE*	325.4	293.76	357.81	413.47	409.05	782.08
*MAE*	0.2608	0.2255	0.2593	0.2979	0.2894	0.5597
*Residual Prediction Deviation (RPD)*	6.2213	7.5862	6.2466	5.5719	5.3788	2.8806
*Range Error Ratio (RER)*	19.55	23.84	19.63	17.511	16.904	9.053
*Ratio* of *Performance* to *Inter-Quartile distance* (*RPIQ*)	11.735	14.309	11.782	10.51	10.145	5.433
*Bias factor (B_f_)*	0.998	1.003	1.0077	1.009	1.0068	1.01
*Accuracy factor (A_f_)*	1.039	1.035	1.043	1.05	1.049	1.094
*Cohen’s d effect size*	0.007	−0.007	−0.016	−0.02	−0.013	0.0001
***Standardized means difference (theta)***	0.007	−0.007	−0.016	−0.02	−0.135	0.0001
*R-squared (R^2^) index*	0.974	0.982	0.974	0.967	0.965	0.878

**Table 6:** Performance of developed models for TVC (mean—reduced dataset case).

Statistical Index “Mean” (76/8)	PCA/NF	FS/NF	WNN	MLP	SVM	PLSR
*Root mean squared error (RMSE)*	0.5077	0.3851	0.4603	0.5261	0.5169	0.7782
*Mean absolute percentage error (MAPE %)*	6.3304	5.1049	5.6459	6.2491	6.3402	8.9732
*Standard error of prediction (SEP %)*	6.7112	5.0907	6.0844	6.9542	6.8331	10.286
*APE*	50.643	40.839	45.167	49.993	50.721	71.785
*MAE*	0.4716	0.3425	0.3789	0.4480	0.4429	0.6830
*Residual Prediction Deviation (RPD)*	3.6044	4.752	3.976	3.478	3.540	2.351
*Range Error Ratio (RER)*	10.775	14.205	11.885	10.399	10.583	7.03
*Ratio* of *Performance* to *Inter-Quartile distance* (*RPIQ*)	4.4231	5.831	4.878	4.268	4.344	2.885
*Bias factor (B_f_)*	0.984	0.967	0.969	0.956	0.944	0.962
*Accuracy factor (A_f_)*	1.066	1.054	1.06	1.066	1.068	1.096
*Cohen’s d effect size*	0.05	0.107	0.095	0.17	0.216	0.22
***Standardized means difference (theta)***	0.047	0.103	0.091	0.16	0.203	0.189
*R-squared (R^2^) index*	0.912	0.949	0.927	0.905	0.908	0.793

**Table 7:** Performance of developed models for TVC (sd—LOOCV case).

Statistical Index sd (LOOCV)	FS/NF	WNN	MLP	SVM	PLSR
*Root mean squared error (RMSE)*	0.2941	0.3525	0.3898	0.4171	1.2696
*Mean absolute percentage error (MAPE %)*	3.2242	3.6716	4.1344	5.3414	17.103
*Standard error of prediction (SEP %)*	4.1969	5.0294	5.5624	5.9519	18.115
*APE*	270.83	308.41	347.29	448.67	1436.7
*MAE*	0.1904	0.2322	0.2536	0.2969	0.9901
*Residual Prediction Deviation (RPD)*	7.3852	6.1628	5.5723	5.2076	1.7109
*Range Error Ratio (RER)*	23.209	19.368	17.512	16.366	5.3771
*Ratio* of *Performance* to *Inter-Quartile* *distance* (*RPIQ*)	13.93	11.624	10.51	9.8228	3.2273
*Bias factor (B_f_)*	1.0073	1.0115	1.0121	1.007	1.0226
*Accuracy factor (A_f_)*	1.0321	1.0365	1.0404	1.0548	1.1734
*Cohen’s d effect size*	−0.016	−0.028	−0.032	−0.013	0.0001
***Standardized means difference (theta)***	−0.016	−0.028	−0.032	−0.013	0.0001
*R-squared (R^2^) index*	0.981	0.9734	0.9674	0.9627	0.6543

**Table 8:** Performance of developed models for TVC (sd—reduced dataset case).

Statistical Index sd (76/8)	FS/NF	WNN	MLP	SVM	PLSR
*Root mean squared error (RMSE)*	0.3876	0.4907	0.5349	0.5481	1.3425
*Mean absolute percentage error (MAPE %)*	4.0154	5.4016	5.5192	7.1125	16.227
*Standard error of prediction (SEP %)*	5.1237	6.4868	7.0708	7.2453	17.746
*APE*	32.123	43.213	44.153	56.899	129.81
*MAE*	0.3096	0.4087	0.4335	0.4614	1.1043
*Residual Prediction Deviation (RPD)*	4.7211	3.7290	3.4210	3.3387	1.3631
*Range Error Ratio (RER)*	14.114	11.148	10.227	9.9813	4.075
*Ratio* of *Performance* to *Inter-Quartile* *distance* (*RPIQ*)	5.7935	4.576	4.198	4.097	1.6727
*Bias factor (B_f_)*	0.9973	1.0005	1.0015	0.9605	0.9349
*Accuracy factor (A_f_)*	1.041	1.055	1.056	1.0775	1.1843
*Cohen’s d effect size*	−0.002	−0.003	−0.008	0.1026	0.3869
***Standardized means difference (theta)***	−0.002	−0.003	−0.008	0.1032	0.3211
*R-squared (R^2^) index*	0.948	0.9178	0.9024	0.897	0.385

**Table 9:** Performance of various ensemble schemes for TVC (LOOCV case).

Statistical Index Ensemble (LOOCV)	Aver	PLSR	Non-Reg
*Root mean squared error (RMSE)*	0.2220	0.1980	0.1770
*Mean absolute percentage error (MAPE %)*	2.8322	2.3802	2.2914
*Standard error of prediction (SEP %)*	3.1682	2.8246	2.5250
*APE*	237.90	199.93	192.48
*MAE*	0.1704	0.1541	0.1423
*Residual Prediction Deviation (RPD)*	9.7833	10.973	12.275
*Range Error Ratio (RER)*	30.746	34.485	38.577
*Ratio* of *Performance* to *Inter-Quartile distance* (*RPIQ*)	18.453	20.698	23.153
*Bias factor (B_f_)*	1.0061	1.0001	1.0005
*Accuracy factor (A_f_)*	1.0284	1.0241	1.0231
*Cohen’s d effect size*	−0.012	0.0001	−0.0001
***Standardized means difference (theta)***	−0.012	0.0001	−0.0001
*R-squared (R^2^) index*	0.9894	0.9916	0.9966

**Table 10:** Performance of various ensemble schemes for TVC (reduced dataset case).

Statistical Index Ensemble (76/8)	Aver	PLSR	Non-Reg
*Root mean squared error (RMSE)*	0.3048	0.2828	0.2552
*Mean absolute percentage error (MAPE %)*	3.7952	3.8070	3.6817
*Standard error of prediction (SEP %)*	4.0290	3.7381	3.3729
*APE*	30.361	30.455	29.453
*MAE*	0.2700	0.2556	0.2431
*Residual Prediction Deviation (RPD)*	6.0038	6.4710	7.1718
*Range Error Ratio (RER)*	17.948	19.3457	21.4407
*Ratio* of *Performance* to *Inter-Quartile distance* (*RPIQ*)	7.3675	7.9409	8.8008
*Bias factor (B_f_)*	0.9859	0.9819	0.9739
*Accuracy factor (A_f_)*	1.0391	1.0394	1.0384
*Cohen’s d effect size*	0.0425	0.0519	0.0888
***Standardized means difference (theta)***	0.0405	0.0498	0.0847
*R-squared (R^2^) index*	0.9683	0.9727	0.9778

## Data Availability

The data used in this study were provided personally to the corresponding author and are not available.

## References

[1] Gagaoua M., Picard B. (2020). Current Advances in Meat Nutritional, Sensory and Physical Quality Improvement. Foods.

[2] Milford A.B., Le Mouël C., Bodirsky B.L., Rolinski S. (2019). Drivers of meat consumption. Appetite.

[3] Luong N.M., Coroller L., Zagorec M., Membré J.M., Guillou S. (2020). Spoilage of Chilled Fresh Meat Products during Storage: A Quantitative Analysis of Literature Data. Microorganisms.

[4] Ellis D.I., Broadhurst D., Kell D.B., Rowland J.J., Goodacre R. (2002). Rapid and quantitative detection of the microbial spoilage of meat by fourier transform infrared spectroscopy and machine learning. Appl. Environ. Microbiol..

[5] Yang D., Lu A., Ren D., Wang J. (2018). Detection of total viable count in spiced beef using hyperspectral imaging combined with wavelet transform and multiway partial least squares algorithm. J. Food Saf..

[6] Ali A.A., Altemimi A.B., Alhelfi N., Ibrahim S.A. (2020). Application of Biosensors for Detection of Pathogenic Food Bacteria: A Review. Biosensors.

[7] Dissing B., Papadopoulou O., Tassou C., Ersbøll B., Carstensen J., Panagou E., Nychas G.-J. (2013). Using multispectral imaging for spoilage detection of pork meat. Food Bioprocess Technol..

[8] Wojnowski W., Kalinowska K., Majchrzak T., Płotka-Wasylka J., Namieśnik J. (2019). Prediction of the Biogenic Amines Index of Poultry Meat Using an Electronic Nose. Sensors.

[9] Sanchez P.D., Arogancia H.B., Boyles K.M., Pontillo A.J., Mohd Ali M.M. (2022). Emerging non-destructive techniques for the quality and safety evaluation of pork and beef: Recent advances, challenges, and future perspectives. Appl. Food Res..

[10] Munekata P.E.S., Finardi S., de Souza C.K., Meinert C., Pateiro M., Hoffmann T.G., Domínguez R., Bertoli S.L., Kumar M., Lorenzo J.M. (2023). Applications of Electronic Nose, Electronic Eye and Electronic Tongue in Quality, Safety and Shelf Life of Meat and Meat Products: A Review. Sensors.

[11] Nicolaou N., Goodacre R. (2008). Rapid and quantitative detection of the microbial spoilage in milk using Fourier transform infrared spectroscopy and chemometrics. Analyst.

[12] Gowen A.A., O’Donnell C.P., Cullen P.J., Downey G., Frias J.M. (2007). Hyperspectral imaging—An emerging process analytical tool for food quality and safety control. Trends Food Sci. Technol..

[13] Tao F., Peng Y. (2014). A method for non-destructive prediction of pork meat quality and safety attributes by hyperspectral imaging technique. J. Food Eng..

[14] Zheng X., Peng Y., Wang W. (2017). A Nondestructive Real-Time Detection Method of Total Viable Count in Pork by Hyperspectral Imaging Technique. Appl. Sci..

[15] Liu D., Pu H., Sun D.-W., Wang L., Zeng X.-A. (2014). Combination of spectra and texture data of hyperspectral imaging for prediction of pH in salted meat. Food Chem..

[16] Kamruzzaman M., Makino Y., Oshita S. (2016). Rapid and non-destructive detection of chicken adulteration in minced beef using visible near-infrared hyperspectral imaging and machine learning. J. Food Eng..

[17] Edwards K., Hoffman L.C., Manley M., Williams P.J. (2023). Raw Beef Patty Analysis Using Near-Infrared Hyperspectral Imaging: Identification of Four Patty Categories. Sensors.

[18] Miller J.N., Miller J.C. (2018). Statistics and Chemometrics for Analytical Chemistry.

[19] Biancolillo A., Marini F., Ruckebusch C., Vitale R. (2020). Chemometric Strategies for Spectroscopy-Based Food Authentication. Appl. Sci..

[20] Saha D., Manickavasagan A. (2021). Machine learning techniques for analysis of hyperspectral images to determine quality of food products: A review. Curr. Res. Food Sci..

[21] Panagou E., Papadopoulou O., Carstensen J.M., Nychas G.-J. (2014). Potential of multispectral imaging technology for rapid and non-destructive determination of the microbiological quality of beef filets during aerobic storage. Int. J. Food Microbiol..

[22] Liu C., Liu W., Lu X., Ma F., Chen W., Yang J., Zheng L. (2014). Application of multispectral imaging to determine quality attributes and ripeness stage in strawberry fruit. PLoS ONE.

[23] Dissing B.S., Nielsen M.E., Ersbøll B.K., Frosch S. (2011). Multispectral Imaging for Determination of Astaxanthin Concentration in Salmonids. PLoS ONE.

[24] Cruz-Castillo J.G., Ganeshanandam S., Mackay B.R., Lawes G.S., Lawoko C.R.O., Woolley D.J. (1994). Applications of canonical discriminant analysis in horticultural research. Hortscience.

[25] Cozzolino D., Murray I. (2004). Identification of animal meat muscles by visible and near infrared reflectance spectroscopy. LWT Food Sci. Technol..

[26] Jolliffe I.T. (2016). Principal Component analysis: A review and recent developments. Phil. Trans. R. Soc. A..

[27] Rokach L. (2010). Ensemble-based classifiers. Artif. Intell. Rev..

[28] Degenhardt F., Seifert S., Szymczak S. (2019). Evaluation of variable selection methods for random forests and omics data sets. Brief. Bioinform..

[29] Chtioui Y., Bertrand D., Barba D. (1998). Feature selection by a genetic algorithm. Application to seed discrimination by artificial vision. J. Sci. Food Agric..

[30] Yamashita T., Yamashita K., Kamimura R. (2007). A Stepwise AIC Method for Variable Selection in Linear Regression. Commun. Stat. Theory Methods.

[31] König G., Molnar C., Bischl B., Grosse-Wentrup M. Relative Feature Importance. Proceedings of the 2020 25th International Conference on Pattern Recognition (ICPR).

[32] Poona N.K., van Niekerk A., Nadel R.L., Ismail R. (2016). Random Forest (RF) Wrappers for Waveband Selection and Classification of Hyperspectral Data. Appl. Spectrosc..

[33] Lindeman R.H., Merenda P.F., Gold R.Z. (1980). Introduction to Bivariate and Multivariate Analysis.

[34] Mehmood T., Sæbø S., Liland K. (2020). Comparison of variable selection methods in Partial Least Squares Regression. J. Chemom..

[35] Kodogiannis V.S. (2017). Application of an Electronic Nose Coupled with Fuzzy-Wavelet Network for the Detection of Meat Spoilage. Food Bioprocess Technol..

[36] Alshejari A., Kodogiannis V.S. (2017). An intelligent decision support system for the detection of meat spoilage using multispectral images. Neural Comput. Appl..

[37] Kodogiannis V.S., Petrounias I., Kontogianni E. Identification of meat spoilage by FTIR spectroscopy and neural networks. Proceedings of the 2014 International Joint Conference on Neural Networks (IJCNN).

[38] Amina M., Panagou E.Z., Kodogiannis V.S., Nychas G.-J.E. (2010). Wavelet neural networks for modelling high pressure inactivation kinetics of Listeria monocytogenes in UHT whole milk. Chemom. Intell. Lab. Syst..

[39] Panagou E.Z., Mohareb F.R., Argyri A.A., Bessant C.M., Nychas G.J. (2011). A comparison of artificial neural networks and partial least squares modelling for the rapid detection of the microbial spoilage of beef fillets based on Fourier transform infrared spectral fingerprints. Food Microbiol..

[40] Rosnow R.L., Rosenthal R., Rubin D.B. (2000). Contrasts and correlations in effect-size estimation. Psychol. Sci..

[41] Amina M., Kodogiannis V.S., Petrounias I.P., Lygouras J.N., Nychas G.-J.E. (2012). Identification of the Listeria monocytogenes survival curves in UHT whole milk utilising local linear wavelet neural networks. Expert Syst. Appl..

[42] Han F., Huang X., Aheto J.H., Zhang X., Rashed M.M.A. (2022). Fusion of a low-cost electronic nose and Fourier transform near-infrared spectroscopy for qualitative and quantitative detection of beef adulterated with duck. Anal. Methods.

[43] Teixeira Dos Santos C., Páscoa R., Porto P., Cerdeira A., González-Sáiz J., Pizarro C., Lopes J. (2018). Raman spectroscopy for wine analyses: A comparison with near and mid infrared spectroscopy. Talanta.

[44] Kodogiannis V.S., Alshejari A. (2014). An adaptive neuro-fuzzy identification model for the detection of meat spoilage. Appl. Soft Comput..

[45] Lee D.S., Lee M.W., Woo S.H., Kim Y., Park J.M. (2016). Nonlinear dynamic partial least squares modelling of a full-scale biological wastewater treatment plant. Process Biochem..

[46] Wijaya D.R., Afianti F., Arifianto A., Rahmawati D., Kodogiannis V.S. (2022). Ensemble machine learning approach for electronic nose signal processing. Sens. Bio-Sens. Res..

[47] Zhang Y., Liu J., Shen W. (2022). A Review of Ensemble Learning Algorithms Used in Remote Sensing Applications. Appl. Sci..

